# 2262 Is less more? Examining the relationship between food assistance generosity and childhood obesity

**DOI:** 10.1017/cts.2018.290

**Published:** 2018-11-21

**Authors:** Megan M. Reynolds, Melanie Beagley, Ashley M. Fox, Ming Wen, Michael W. Varner, Ken R. Smith

**Affiliations:** 1 The University of Utah School of Medicine; 2 The University of Utah; 3 University at Albany, State University of New York

## Abstract

OBJECTIVES/SPECIFIC AIMS: In combination with 3 waves of individual-level data on children age 5–18 from the Panel Study of Income Dynamics, we exploit exogenous variation at the level of the state to determine whether SNAP generosity modifies the effect of SNAP participation on overweight/obesity status. We do so using a newly created and powerful data set including information on state-level SNAP generosity between the years 1996 to 2011. METHODS/STUDY POPULATION: Data and sample. We drew individual-level data from the Child Development Supplements of the Panel of Income Dynamics (PSID), a nationally representative longitudinal study gathering data since 1968 on US individuals and the families in which they reside. Aged 0–12 years in 1997, these children of PSID sample members were surveyed roughly every 5 years through 2007. The total number of observations over the study period is just over 8093, representing 3563 children. We drew state-level data from the State Welfare Generosity Index. This is a decomposable index of State welfare generosity capturing state policy variation across 4 programs (TANF, SNAP, Unemployment Insurance and Medicaid/CHIP) and 2 dimensions (eligibility requirements and benefit levels). Measures. Child weight status was determined using the Center for Disease Control (CDC) body mass index (BMI)-for-age gender-specific growth charts: underweight (BMI <5th percentile), healthy weight (BMI >5th percentile and BMI <85th percentile), overweight (BMI >85th percentile and BMI <95th percentile) or obese (BMI >95th percentile). From this, we constructed an indicator for overweight/obese Versus normal or underweight status. SNAP participation is a dichotomous indicator based on the head-of-households or their spouses reported receipt of SNAP benefits during the previous calendar year from the interview. SNAP generosity is scored on a scale of 0–100, with more generous states receiving higher scores than less generous states. Covariates include sex, race, age, head-of-household years of education and a continuous measure of household income adjusted for family size. Estimation techniques. We merged the child, parent/caregiver, family and main PSID files to obtain the most comprehensive data on each sample child. We first generated, descriptive statistics for the Wave 1 sample of 3563 children. We then present the mean, standard deviation and the ratio of the 2 (coefficient of variation) for state-level variables. We present χ^2^ tests of difference for non-SNAP compared to SNAP participants in terms of overweight/obesity, and pairwise correlation coefficients among the 3 state-level variables. Next, we conducted a series of simple and multivariate logistic regressions estimating the odds of being overweight or obese. As we are assessing the risk of adverse weight status, those of normal or underweight status are the reference group for all regression analysis. Because height and weight reports are known to be unreliable below the age of 5, regression analyses impose an age restriction of greater than 5 years old. We include adjustment for the clustered nature of data. RESULTS/ANTICIPATED RESULTS: The individual-level statistics indicate that roughly one-third of the CDS sample is overweight or obese at Wave I in 1997. About a fifth of them live in families receiving SNAP. The mean SNAP generosity score is 10 on a possible range of 0 to 1 (observed range of 0.037 to 0.290 not shown). Variation across state-years is greatest for the SNAP participation variable, as reflected by the coefficient of variation. In the period 1997–2007, the proportion of children who are overweight or obese is 5% higher among those in families receiving SNAP program benefits than among those in families not receiving SNAP benefits. Similarly, SNAP participation is positively, moderately and significantly (with an α of 0.05) correlated with overweight/obesity. Examines the relationship between overweight/obesity and the SNAP measures using individual-level data on overweight/obesity and SNAP participation and state-level data on SNAP generosity. Model 1 estimates and exponentiates the log odds of overweight/obesity based on individual-level SNAP participation. Model 2 does the same using state-level SNAP generosity as the predictor. Results indicate that both variables are positively associated with a child’s chance of being overweight/obese. But only in the case of SNAP participation is the SNAP variable statistically significant. Children living in families receiving SNAP benefits are more likely to be overweight/obese by a factor of 1.23. A set of potential confounders to the analysis and tests for interaction effects between SNAP participation and SNAP generosity (Model 3). Controlling for a variety of demographic and socio-economic factors, the positive effect of SNAP participation on overweight/obesity is rendered negative. The nonsignificant effect of SNAP generosity remains. In Model 3, the interaction effect for SNAP participation and generosity is positive and marginally significant. This suggests that the generosity of benefits changes the basic relationship between SNAP participation and overweight/obesity among children in families receiving benefits. To help convey the meaning of this coefficient, we generated marginal effects of SNAP participation based on SNAP generosity, setting all covariates equal to their means. This figure shows a small negative effect of SNAP participation at the lowest levels of generosity (a score of around 4, the sample minimum). This negative effect crosses 0 at a score of around 12, then becomes positive. The magnitude of the positive effect grows up to the sample max (index=28), although with widening confidence intervals. DISCUSSION/SIGNIFICANCE OF IMPACT: The focal interest of this study lies in the potential interaction effect between SNAP generosity and SNAP participation on overweight/obesity. Although the effects were only marginally significant, we find that SNAP generosity does interact with SNAP participation. More specifically, the effects of SNAP participation appear negative at lower levels of generosity, becoming positive as generosity scores exceed the sample mean (index=10). In other words, state-level SNAP generosity appears to exacerbate the adverse effects of SNAP participation on overweight/obesity. Although we submit that our current findings contribute to the literature on the SNAP-health link, we intend to strengthen our analysis in several ways. First, we will fit models that exploit the strengths of the PSID and the welfare generosity index in terms of causal inference. We will use fixed effects models to control not only for potential unobserved confounders related to the child but also observable baseline characteristics. Leveraging the fact that PSID samples up to 2 children from each family, we will further refine our estimates towards a causal interpretation with the use of sibling fixed effects, in which we additional account for unmeasured time-invariant family-level variables that encapsulate a variety of factors including learned behaviors, cultural influences, genetic predispositions that contribute to child health outcomes. Second, research has clearly shown that compared with higher-SES individuals, lower-SES individuals have higher BMI regardless of welfare program participation. These selection effects are addressed somewhat by the PSID’s intentional over-representation of low-income individuals. But we can much more convincingly address these potential problems with endogeneity by refining our analyses to compare SNAP participants to SNAP-eligible nonparticipants, thereby isolating the effect of the SNAP “treatment.” Lastly, we intend to include a wide array of state-level covariates that may be related to our independent and dependent variables of interest, such as poverty rate, unemployment rate, and racial/ethnic composition.

